# “Cerebellar Challenge” for Older Adults: Evaluation of a Home-Based Internet Intervention

**DOI:** 10.3389/fnagi.2017.00332

**Published:** 2017-10-27

**Authors:** Zoe Gallant, Roderick I. Nicolson

**Affiliations:** Department of Psychology, University of Sheffield, Sheffield, United Kingdom

**Keywords:** declarative memory, cerebellum, hippocampus, sensorimotor, balance, vestibular stimulation, functional networks

## Abstract

There is converging evidence that maintenance of function in the multiple connectivity networks involving the cerebellum is a key requirement for healthy aging. The present study evaluated the effectiveness of a home-based, internet-administered “cerebellar challenge” intervention designed to create progressive challenges to vestibular function, multi-tasking, and dynamic coordination. Participants (*n* = 98, mean age 68.2, SD 6.6) were randomly allocated to either intervention (the cerebellar challenge training for 10 weeks) or no intervention. All participants undertook an initial series of pre-tests, and then an identical set of post-tests following the intervention period. The test battery comprised five suites of tests designed to evaluate cognitive-sensori-motor-affective functions, including Physical Coordination, Memory, Language Dexterity, Fluid Thinking and Affect. The intervention group showed significant pre- to post improvements in 9 of the 18 tests, whereas the controls improved significantly on one only. Furthermore, the intervention group showed significantly greater improvement than the controls on the “Physical Coordination” suite of tests, with evidence also of differential improvement on the Delayed Picture Recall test. Frequency of intervention use correlated significantly with the improvement in balance and in peg-moving speed. It is concluded that an internet-based cerebellar challenge programme for older adults can lead to benefits in balance, coordination and declarative memory. Limitations and directions for further research are outlined.

## Introduction

The brain and body form a complex, self-regulating system capable of coping with a range of environmental and cognitive challenges, together with the pervasive, age-related progressive impairment in function of many system components. In this article we develop the perspective that the functional networks involving the cerebellum represent a significant part of the degradation in aging. We then briefly review the many interventions that have proved efficacious with older adults, noting the current consensus that multi-component systems designed to maintain a progressive challenge appear to have greater effect than single component systems. On theoretical grounds we argue that interventions designed around “cerebellar challenge”, combining coordinative exercise with cerebellar stimulation, should prove particularly effective. We finish by presenting an evaluation of an internet-based cerebellar challenge system, Zing, in terms of its effectiveness compared with a life-as-usual control group.

Traditional approaches to the causes of cognitive decline with aging considered primarily the frontal lobes (Jackson, 1958; Dempster, 1992; Greenwood, 2000). Over the past three decades there has been an explosion of research on all aspects of aging. Early this century extensive research was undertaken on changes in brain structure with aging (Raz et al., 2005), genetics (Deary et al., 2004; Erraji-Benchekroun et al., 2005), together with risk factors including increased white matter (Bartzokis, 2004; Head et al., 2004; Westlye et al., 2010; Sexton et al., 2014); excess homocysteine (Schafer et al., 2005) and reductions in dopamine and acetylcholamine neurotransmitters (Castner and Goldman-Rakic, 2004; Sarter and Bruno, 2004; Erixon-Lindroth et al., 2005).

Following these discoveries, arguably the greatest recent development has been the change of emphasis from these individual components and processes of the aging brain to consideration of the brain as a whole system. A major recent development in cognitive neuroscience has been the development of techniques for determining functional connectivity (Greicius et al., 2003; Fox et al., 2005; Buckner et al., 2008), and the consequent identification of a range of intrinsic networks (Yeo et al., 2011). The approach has great potential for characterizing the connectivity problems that affect brain function. A recent review (Bamidis et al., 2014) highlights the key role of connectivity changes in brain aging, and its implications for assessment and intervention.

It is notable that the cerebellum is also involved in seven of the major intrinsic networks (Buckner et al., 2011; Bernard et al., 2012; Kipping et al., 2013). It is therefore particularly interesting that circuits involving the cerebellum are strongly affected by age (Seidler et al., 2010; Balsters et al., 2013; Bernard et al., 2013; Humes et al., 2013; Bernard and Seidler, 2014; Koppelmans et al., 2015). Furthermore, it appears that the pattern of cerebellar degeneration with age in healthy adults is analogous to that shown by cerebellar patients (Hulst et al., 2015).

It is long established that there are major declines with age in sensory function (Humes et al., 2013; Wayne and Johnsrude, 2015; Roberts and Allen, 2016), in motor function (Seidler et al., 2010) and proprioceptive function (Goble et al., 2009). The cerebellum is centrally involved in sensorimotor processing (Chadderton et al., 2004, 2014; Ramakrishnan et al., 2016) and the involvement of the cerebellum in cognitive function is now fully established (Balsters et al., 2013; Mariën et al., 2014), as are direct, two-way links between the cerebellum and not only motor cortex but also prefrontal and posterior parietal cortex and the basal ganglia (Strick et al., 2009; Bostan et al., 2013).

Taken together, these results converge on the hypothesis (Bernard and Seidler, 2014) that the cerebellum—given its pervasive connectivity, its involvement in multiple sensory, cognitive and motor circuits; and its central role in adapting to internal changes—may be a critical component in the system degradation with age. This new conceptualization offers the promise that interventions designed to maintain or enhance cerebellar function may alleviate the affects of aging on sensori-motor-cognitive performance.

There are many successful interventions for alleviating age-related decline. A recent review (Ballesteros et al., 2015) focused on three modes of intervention: physical activity, computerized cognitive training and social enhancement and concluded that although single domain interventions were effective the simultaneous training of both cognitive and physical domains offers a greater potential on daily life functioning. One of the key problems identified by the authors was the issue of how to combine different interventions and how to evaluate their effectiveness. The systems approach to healthy aging provides a theoretical perspective on this issue, suggesting that if a major cause if impairment is functional loss in the intrinsic connectivity networks, the optimal intervention should target function in the network as a whole, rather than individual components thereof.

Computerized cognitive training (CCT) approaches, using computer programs to boost core cognitive capabilities such as working memory, speed of processing and visual attention have proved highly effective in some studies, but less so in others. Systematic reviews of brain training programmes with older adults (Gross et al., 2012; Kueider et al., 2012) concluded that computerized training is an effective, less labor intensive alternative to cognitive training. In contrast, a recent analysis (Lampit et al., 2014) concluded that the overall effect size of CCT vs. control was small and statistically significant for nonverbal memory, verbal memory, working memory, processing speed, and visuospatial skills but not for executive functions and attention. A meta-analysis for younger groups (Melby-Lervåg and Hulme, 2013) concluded that WM programs produced reliable short-term improvements in WM skills but that the effects were “*short-term, specific training effects that do not generalize”*.

One of the clear limitations, from a systems view, both of CCT and of direct brain stimulation, is that the intervention is artificial, and isolated from the physical or mental activities involved in normal system functionality. There is strong evidence that natural activities, such as exercise, can improve not only physical fitness but also mental fitness, and even stimulate the growth of new brain neurons and connections (Hillman et al., 2008; Höetting and Röeder, 2013; Kirk-Sanchez and McGough, 2014). An innovative approach, the Long-Lasting Memories intervention, which combines both exercise and CCT approaches (“exergaming”) was shown to have beneficial effects for healthy older adults and for those with Mild Cognitive Impairment (MCI; González-Palau et al., 2014).

A recent discovery has been the differential effects of cardiovascular, high intensity, exercise and “co-ordinative exercise” such as balance training or tai-chi. There is strong evidence that exercise can potentiate the brain for new learning, with coordinative balance exercises leading to neural growth in the hippocampus—a core structure for explicit learning and memory (Niemann et al., 2014)—and also in the cerebellar-cortical loop (Burciu et al., 2013)—a core network for implicit learning and coordination. A further study (Nascimento et al., 2014) concluded that multimodal physical exercise was effective in reducing pro-inflammatory cytokines and in improving brain-derived neurotrophic factor (BDNF) peripheral levels, with positive reflexes on cognition in elderly individuals with MCI.

Recent studies of the effects of exercise on rat brains (Kellermann et al., 2012; Abel and Rissman, 2013) reveal strong effects on epigenetic changes and changes in the cerebellar Purkinje cells following a rat vestibular training exercise (Lee et al., 2015). There is also evidence that BDNF is expressed in the cerebellum following environmental enrichment for rats (Angelucci et al., 2009; Vazquez-Sanroman et al., 2013). Of particular interest, there is evidence (though sparse) that Quadrato exercise (like Tai Chi) led to increased creativity and changes in gray matter and white matter in the cerebellum (Ben-Soussan et al., 2015).

There have also been detailed neuroimaging studies of interventions for special groups. Daily clinic-based balance training for 2 weeks in cerebellar patients and age-matched healthy controls (Burciu et al., 2013) led to enhanced balance performance in the patients, with associated increased gray matter volume in the dorsal premotor cortex and within the cerebellum for both groups. A 6 week balance-training study with Parkinson’s patients and healthy controls (Sehm et al., 2014) led to improved balance which was maintained for the following year, together with increased gray matter in the hippocampus for the controls and in several brain regions for the patients.

Of particular interest regarding functional connectivity, two recent studies with older adults with MCI have established functional connectivity changes following 8 week interventions. Klados et al. (2016) established that the Long Lasting Memories intervention led to increased beta-band EEG activity (reflecting increased bilateral connections in the occipital, parietal, temporal and prefrontal regions) after the intervention. Chirles et al. (2017) undertook a “walking exercise” intervention, and established that following the intervention the MCI group showed increased connectivity in 10 regions spanning frontal, parietal, temporal and insular lobes, together with the cerebellum.

In summary, current neuroimaging and behavioral research appears to be converging to a view that: (i) a systems approach to aging is the most promising framework for understanding the degradation in multiple functions with age; (ii) there is extensive evidence that the cerebellum is one of the key structures affected, and the multiple intrinsic connectivity networks linking the cerebellum with other brain and body structures may well mediate many of the actual deficits shown; (iii) “single system” interventions can be effective, but generally it is better to have multiple domain interventions; (iv) a range of interventions, from coordinative exercise to direct vestibular stimulation are likely to have beneficial effects on cerebellar function.

The above considerations informed the design of the current study. We wished to evaluate the effectiveness of a novel internet-based “vestibular stimulation” intervention, the Zing intervention^1^. This intervention was originally developed to tune up the coordination abilities of top sporting performers, using a series of graded exercises designed specifically to improve three performance dimensions: sensorimotor coordination, eye movement control and dual tasking. However, extensive feedback had suggested that the programme was valuable for many average performers. Consequently the system was embedded in an internet-based “game” format designed to challenge and stimulate the user to keep improving their performance. Zing Performance offer a number of courses specifically tailored to each individual user, with applications in sporting areas, organizational development, in education.

The Zing system involves a series of graded activities on three dimensions—dynamic activity (patterned movement sequences), focus activity (developing the ability both to concentrate and to “dual task”) and stability activity (coordinative balance). Underpinning the approach is the technique of vestibular stimulation. Rather than cardiovascular exercise, which is designed to have energetic use of highly practised routines, or even coordinative balance such as tai-chi, which does involve learning new actions, vestibular activities are designed to cause abnormal input for the vestibular system, for example by requiring the user to put their head on one side while undertaking tasks. This presents the vestibular system, and the cerebellum, with an immediate challenge, requiring activation of many circuits to cope with the ensuing proprioceptive feedback.

**Figure 1:**
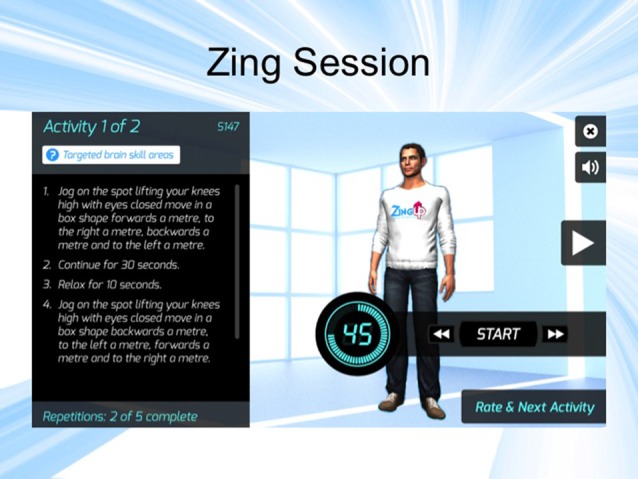
Sample screen from the Zing intervention. This is an example of a screen from the focus strand, at a low level and aimed at developing dual tasking ability.

A typical course lasts 6 months and is composed of daily physical activities and digital video games. An example of a low level focus activity (at the time of the study) is given in Figure 1. A video is also available for each activity.

The Zing platform therefore provides a user-orientated, motivating framework for delivering a cost-effective cerebellar challenge intervention that satisfies the criteria that have emerged for multimodal, challenging interventions in older adults.

We undertook the study to investigate whether internet-based approaches can indeed be an effective and popular method for older adults, and designed an 8 week intervention. It is important to highlight that although this is a Randomized Control Trial (RCT) study, in that there was a control condition and allocation to condition was random, it is not a full RCT, for which an active intervention condition, matched in time and form to the Zing intervention, would need to be used to counter placebo-type effects. Our view is that this non-equivalent–control RCT (NEC-RCT) design is appropriate for a user-centered trial that has the underlying question “If participants undertake the intervention, will it help them, and, if so, in what ways?” We are not investigating the theoretical issue—is intervention A more effective than intervention B, and if so, why? Each design has its strengths and weaknesses. For the purpose of evaluating whether a low-cost, home-based intervention might be beneficial compared with life-as-usual, the NEC-RCT design is the appropriate one.

A limitation of many previous intervention studies is that the set of tests used from pre-intervention to post-intervention focus on a limited range of performance measures. As noted above, there is reason to expect that a cerebellar challenge intervention might lead to changes in both the sensorimotor domain and in the cognitive domain. There is also longstanding evidence that the cerebellum is involved in emotional processing (Schmahmann and Sherman, 1997), with emerging evidence regarding its involvement in processing emotional salience (Styliadis et al., 2015; Adamaszek et al., 2017). There is also evidence that the resting state networks involving the cerebellum are associated with differences in crystallized intelligence (Pezoulas et al., 2017). Consequently we designed a battery of simple tasks designed to probe sensorimotor, performance, cognitive performance, emotional state and nonverbal reasoning.

The design allows the following hypotheses to be evaluated.

Hypothesis 1. Improvements in balance and sensorimotor coordination. This is the primary applied hypothesis, directly related to attempting to boost balance performance and thus decrease the danger of falling. One in three people of 65 fall at least once per year, with the incidence rising to one half of those over 80 years old (Todd and Skelton, 2004). Falls are a major cost to elderly people and to national health services, estimated to account for 21% of the Dutch health service costs for injuries (Hartholt et al., 2011). Hypothesis 1 states that Zing training will lead to significant improvements in sensori-motor coordination especially balance: (a) for each individual compared with their pre-training; (b) that the intervention group will improve significantly more than a control, no intervention, group.

Hypothesis 2. “Hippocampal” improvements. This hypothesis is derived from the research showing the benefits of coordinative balance training for hippocampal function. Hypothesis 2 states that Zing training will lead to significant improvements in “declarative memory” performance: (a) for each individual compared with their pre-training; (b) that the intervention group will improve significantly more than a control, no intervention, group.

Hypothesis 3. Improvement Specificity. Despite the emerging evidence of cerebellar involvement in affective processing, we would expect any such changes to be of secondary importance in terms of affective state. Consequently, although Zing training may lead to significant “transfer” to other areas, including language, affect and fluid reasoning, any such effects will be minor compared with the specific improvements in hippocampal and sensorimotor skills.

## Materials and Methods

### Participants

Ninety-eight volunteers (30 male, 68 female) aged 50–85 (mean 68.2, SD 6.6) were recruited through advertisements in local newspapers, churches and social groups. An advert also went out on the University of the Third Age Sheffield website. Participants were all without a known diagnosis of dementia. The ethics committee of the Department of Psychology, University of Sheffield, approved the study. Participants gave fully informed prior consent. They were also informed that their information would be anonymised and kept securely. They were also informed that they could withdraw from the study at any time without needing to give any reason. All participants were healthy older adults.

### Design

The aim of this study was to test the effectiveness of vestibular stimulation on physical and mental function. Therefore, a repeated measures design was used. Participants were asked to complete a baseline set of tests at the University of Sheffield Department of Psychology before taking part in the 8 week Zing intervention at home. They were then asked to return to the department for a repeat of the baseline tests.

### Test Battery

The same tests were used both pre and post-test. While there may be some practice effects here, it would be expected that this would affect both groups equally, and therefore any relative difference in the intervention group’s performance is likely to be attributable to the exercises.

We wished to evaluate changes in all the core physical, mental and affective domains, using simple but normed tests where possible. We based the battery on the Dyslexia Adult Screening Test (Fawcett and Nicolson, 1998), which covers the majority of the necessary tests in a 30 min package. We constructed a battery of 14 tests, divided into five suites. Suite 1 was for Physical Coordination and comprised the DAST balance test, the two Purdue pegboard (Tiffin and Asher, 1948) tests (Peg Moving and Peg Assembly), and the DAST writing (copying) test. Suite 2 investigated memory. It included two tests of working memory, the DAST backwards digit span test and the South Yorkshire Ageing Study (Tarmey, 2012) Spatial Memory test which determines spatial memory span for non-verbalizable pictures presented in one of eight locations. There was one declarative memory test, the South Yorkshire Ageing Study (Tarmey, 2012) Picture Memory test which assesses recall for a set of 20 pictures of common objects, presented sequentially for 1 s, including both immediate recall and delayed recall after 20 min. The Language Suite comprised the DAST Rapid Naming, Phonological Processing, Reading, Nonsense Passage and Spelling tests. The Fluid Reasoning suite comprised the DAST Nonverbal Reasoning, Semantic Fluency and Verbal Fluency tests. Finally two tests of affect were administered: the Beck Depression Inventory (BDI; Beck et al., 1996) and the Authentic Happiness Inventory (Seligman, 2002).

### Intervention Training

Participants were required to undertake a minimum of 8 weeks and maximum of 10 weeks balance and sensorimotor coordination training using an online set of activities. These were provided by Zing Performance Ltd., and were designed specifically to stimulate brain regions involved in coordinative balance. Initially, participants had to undergo an assessment to determine their strengths and weaknesses. After this a 30 day programme was set for them, specifically designed to target their biggest needs. Participants were required to do two exercises a day, before rating how difficult they found that particular activity. A screen shot is shown in Figure 1 below. Each week, three exercises were assigned, with two of the three appearing each day. After 30 days, participants were reassessed before continuing onto unit two. It should be noted that a full Zing 360 session programme is designed for 6 months, with two sessions per day. Consequently this study is very much shorter than intended by the Zing designers.

## Results

Data were converted to standard scores (mean 100, SD 15) to allow direct comparison across tasks. Where possible, population norms and standard deviations were used to normalize test scores. The population norm for age 55+ was used for all participants, irrespective of age, to represent absolute performance rather than age-adjusted performance.

### Effect Sizes

In order to facilitate comparison of the improvements (or otherwise) in performance from pre-test to post-test, effect sizes were calculated using the formula ES = (post-test − pre-test) / SD (all groups on pre-test), which is a form of Cohen, 1988 applied to change analysis. No change would result in an effect size of 0, whereas a score of +1.0 indicates a change of one standard deviation unit. Cohen (1988) suggests that effect sizes of 0.8, 0.5 and 0.2 be labeled large, medium and small, respectively.

**Figure 2:**
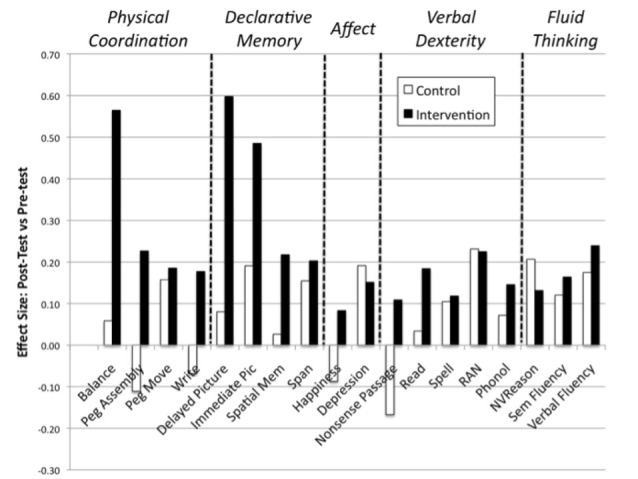
Effect sizes for the variables of interest.

Effect sizes for the two groups are shown in Figure 2. Tests have been grouped in order of the hypotheses. The Physical Coordination suite—postural stability, peg moving, peg assembly and handwriting speed are on the left, then the Memory Suite—immediate picture recall, delayed picture recall, immediate spatial memory and immediate verbal memory. Group 3 include the affect measures—the BDI (reverse scored such that higher means less depressed) and the Authentic Happiness Index. The remaining tests are the Language Suite and the Fluid Thinking Suite but were not predicted to be affected by the intervention.

### Correlations with Zing Usage

Next correlational analyses were undertaken utilizing data collected automatically on “compliance” for the Zing group. Of the 53 participants allocated to the Zing group, 38 completed at least 40 sessions, as requested, but the differential uptake allowed us to investigate the effects both of frequency of Zing use (sessions per week) and the duration (number of weeks). For the frequency of Zing use significant correlations were found for peg movement (*r* = 0.31, *p* < 0.05), and for postural stability (*r* = 0.303, *p* < 0.05). A significant correlation with number of weeks of the intervention occurred only for nonverbal reasoning (*r* = 0.288, *p* < 0.05).

### Within-Group Statistical Tests

Inferential statistical tests were then undertaken for the 16 tests within the five suites of tests. First repeated measures multivariate analyses of variance were undertaken for each suite separately on the data for pre-test and post-test for each test within the suite.

For the control group, none of the set of MANOVAs approached significance. In fact the only individual comparison to reach the uncorrected 0.05 significance level was for peg moving (*F*_(1,43)_ = 6.26, *p* = 0.016).

For the Zing group, the MANOVA analyses of the change from pre-test to post-test were highly significant for the suites for Physical Coordination, for Declarative Memory, for Language, and for Fluid Thinking (*F*_(1,47)_ = 22.95, *p* < 0.001; *F*_(1,52)_ = 10.71, *p* = 0.002; *F*_(1,52)_ = 15.99, *p* < 0.001; *F*_(1,52)_ = 5.72, *p* = 0.020 respectively), whereas there was no difference for the Affect suite. It is not sensible to undertake a Bonferroni correction for multiple comparison when all comparisons are significant in the same direction (Moran, 2003), and consequently uncorrected probabilities are reported. The changes for Balance, Peg Assembly and Peg Movement were significant (*F*_(1,50)_ = 14.07, *p* < 0.001; *F*_(1,51)_ = 5.53, *p* = 0.023; *F*_(1,50)_ = 4.10, *p* = 0.048 respectively). The improvements for Delayed Picture Recall, Immediate Picture Recall and Memory Span were also significant (*F*_(1,52)_ = 14.44, *p* < 0.001; *F*_(1,52)_ = 15.41, *p* < 0.001; *F*_(1,52)_ = 4.20, *p* = 0.046 respectively). Two of the improvements for nonsense passage reading, 1 min reading, rapid naming and spelling were significant [*F*_(1,52)_ = 3.72, *p* = 0.059; *F*_(1,52)_ = 6.28, *p* = 0.015; *F*_(1,52)_ = 4.73, *p* = 0.034; *F*_(1,52)_ = 3.28, *p* = 0.076 respectively). The improvement for verbal fluency was also significant (*F*_(1,52)_ = 5.13, *p* = 0.028).

### Between-Group Statistical Tests

Finally, in the most stringent test of the changes, a series of multivariate 2-factor analyses of variance was undertaken, with the independent groups factor being the group (Zing vs. Control) and the repeated measure being time-of-test (pre-test vs. post-test. Manovas were undertaken separately for each of the five suites (see Table 1). For the MANOVA entry, only the key statistic, the interaction term between time of test (pre vs. post) and Group is reported. A significant interaction would typically indicate that the Intervention led to a significant difference between groups at post-test whereas performance at pre-test was equivalent.

**Table 1:** Multivariate and univariate analyses of variance for the variables of interest.

**(1) Physical coordination**	Manova: *F*_(1,87)_ = 9.47, *p* = 0.003
Postural stability	*F*_(1,89)_ = 5.24, *p* = 0.024, *η*^2^ = 0.056
Peg assembly	*F*_(1,92)_ = 4.36, *p* = 0.040, *η*^2^ = 0.045
Peg move	*F*_(1,94)_ = 0.03, *p* = 0.864, *η*^2^ = 0.000
Writing	*F*_(1,92)_ = 0.10, *p* = 0.754, *η*^2^ = 0.001
**(2) Declarative memory**	Manova: *F*_(1,93)_ = 1.09, *p* = 0.300
Delayed picture recall	*F*_(1,93)_ = 4.58, *p* = 0.035, *η*^2^ = 0.047
Immediate picture recall	*F*_(1,93)_ = 1.78, *p* = 0.185, *η*^2^ = 0.019
Spatial memory	*F*_(1,93)_ = 1.05, *p* = 0.308, *η*^2^ = 0.011
Verbal memory span	*F*_(1,93)_ = 0.15, *p* = 0.703, *η*^2^ = 0.002
**(3) Language**	Manova: *F*_(1,93)_ = 2.57, *p* = 0.113
Nonsense passage reading	*F*_(1,93)_ = 4.20, *p* = 0.043, *η*^2^ = 0.043
One minute reading	*F*_(1,93)_ = 1.72, *p* = 0.193, *η*^2^ = 0.018
Rapid naming	*F*_(1,93)_ = 0.01, *p* = 0.933, *η*^2^ = 0.000
2 min spelling	*F*_(1,93)_ = 0.01, *p* = 0.979, *η*^2^ = 0.000
Spoonerisms	*F*_(1,93)_ = 0.13, *p* = 0.715, *η*^2^ = 0.001
**(4) Fluid thinking**	Manova: *F*_(1,93)_ = 0.06, *p* = 0.813
Nonverbal reasoning	*F*_(1,93)_ = 0.11, *p* = 0.742, *η*^2^ = 0.001
Semantic fluency	*F*_(1,93)_ = 0.06, *p* = 0.805, *η*^2^ = 0.000
Verbal fluency	*F*_(1,93)_ = 0.32, *p* = 0.571, *η*^2^ = 0.003
**(5) Affect**	Manova: *F*_(1,93)_ = 0.99, *p* = 0.755
Authentic happiness index	*F*_(1,93)_ = 2.38, *p* = 0.127, *η*^2^ = 0.025
Beck depression inventory	*F*_(1,94)_ = 0.02, *p* = 0.890, *η*^2^ = 0.001

It may be seen that the only suite returning a significant MANOVA result was the Physical Coordination suite. For each of the four tests a univariate two factor mixed measures analysis of variance was undertaken, with the within-group variable being time-of-test (pre-intervention vs. post-intervention) and the between-group variable being group (intervention vs. control). Significant (uncorrected) interactions—all reflecting greater improvement for the intervention group than the control group—were obtained for postural stability and for peg assembly. By contrast, there were no differences for peg moving speed or writing speed.

The MANOVA results for the other four suites of tests were not close to significance. Uncorrected significant differences were obtained for Delayed Picture Memory and for Nonsense Passage Reading.

### Correlations with Age

Finally, correlations with age were calculated. Significant correlations were found for performance on the majority of tests, with correlations between age and each dependent variable in descending order being −0.47 (Nonverbal reasoning), −0.40 (peg assembly), −0.38 (immediate picture memory), −0.35 (immediate picture memory), −0.34 (writing), −0.28 (spatial memory), −0.27 (semantic fluency), −0.26 (postural stability) and −0.26 (spelling). Correlations between age and the amount of improvement for the Zing group were also calculated. Few correlations were significant, with only peg assembly (−0.37) being more extreme than −0.25.

## Discussion

The primary issue addressed by this study was whether a home-based cerebellar challenge internet-administered intervention was feasible for use with older adults and, if used, whether it would result in better balance, and hence reduce danger of falling (Hypothesis 1). A secondary, theoretical issue, was whether the intervention might also improve cognitive functions previously found to be improved by coordinative balance training (Hypothesis 2).

A set of five “suites” of tests was applied before and after the intervention, allowing comparison with a “life as usual” control group. Comparing individual performances across the intervention period, the control group performance remained roughly constant, with no significant change for 17 of the 18 tests. By contrast, the intervention participants showed significant improvement in their scores for 9 of the 18 tests administered, with only the tests of the Affect suite showing no significant multivariate improvement.

Furthermore, a series of two factor multivariate analyses of variance revealed that the intervention group improved significantly more than the control group on the Physical Coordination suite, but not on the memory suite, the affect suite, the language suite or the fluency suite.

Hypothesis 1 is therefore clearly supported. Not surprisingly—but crucial for applied purposes—the intervention group did improve significantly on balance compared both with their own pre-intervention performance and with the control group’s change in balance over the period of the study. There was also transfer of this training effect to manual dexterity (as indicated by the “peg assembly” task).

Clear support is also provided for Hypothesis 2, though the effect is masked by the inclusion of tests of working memory and declarative memory within the memory suite. It is clear from the effect sizes and the between-groups anova data that there was a significant benefit for the Zing group for the tests of declarative memory (especially the key task of delayed picture recall, which is more akin to a realistic memory use task) but not for the tests of working memory. It is therefore legitimate to infer that, consistent with the literature on the benefits of balance training on hippocampal function, there was transfer of this benefit to declarative memory in the delayed picture recall condition (Hypothesis 2).

The specificity of the differential benefit findings to the hypotheses suggests strongly that the changes are not a practice, placebo or Hawthorne effect (Hypothesis 3). Given the null effect on affect, we examined the individual scores on the BDI in order to investigate whether participants more at risk of depression showed differential effects. Of the four participants in the intervention group classifiable as at least mildly depressed, one showed marked improvement. However, the 2 participants in the non-intervention group initially showing mild depression also showed marked improvement. We conclude that, at least in this group of heathy older adults, the results do not suggest that there is a direct effect of the cerebellar challenge intervention on affective state.

Of the 52 participants selected for the intervention condition, 38 (73%) completed the requested 40 sessions over 8 weeks. Analyses of the “dose effect” (that is, correlations of performance improvement with number of intervention sessions for the intervention group) revealed significant correlations with intervention frequency for peg movement, reading and balance, with a significant correlation with intervention duration for nonverbal reasoning.

In terms of the participants’ response to the intervention, it should be noted that the Zing platform was a prototype version, not yet publicly available and in the process of substantial development and improvement. A sizeable minority of the Zing participants reported difficulties in accessing the system initially, though subsequently problems were relatively small.

It is important to acknowledge the limitations of this study. First, the population sampled was by no means random, involving respondents to a circular. They should therefore be seen as relatively high functioning and with good self efficacy. Furthermore, they represented a spread of ages, with 2 under 55, 27 aged 55–64, 55 aged 65–74 and 15 aged 75 and over. There was a considerable imbalance between the sexes, with 30 male and 68 female. All were living at home in reasonable health. All of these factors reduce the strength of inferences that can be made regarding generalization to the full population of community based older adults, and highlight the need for further research.

## Conclusion

Prior research has established that home-based balance exercises are among the most cost-effective methods of improving balance ability and hence reducing falls in older adults. Recent developments in cognitive neuroscience have revealed that “coordinative” balance training is likely to have beneficial effects not only on physical coordination but also on hippocampal function. Our theoretical analyses suggested that a multi-component, cerebellar challenge intervention should prove highly effective, combining the effectiveness of coordinative exercise with that of direct cerebellar stimulation, and therefore improving function in the intrinsic connectivity networks involving the cerebellum. The study design did not include brain imaging, and therefore it is not possible to assess directly any underlying neural changes. Furthermore the study design does not permit comparison of the Zing approach with a suitable active control. Nonetheless, the results were encouraging.

The present study is unique in two ways: first we investigated a highly cost-effective internet-based “cerebellar challenge” intervention, “Zing”. Second we investigated physical coordination, mental coordination, language, fluid thinking and affect using a specially developed battery of tests. Significant benefits (comparing initial performance with post-intervention performance) were found for the intervention group on the majority of the tests, excluding only those for affect. Furthermore, significantly greater improvements were found for the intervention group (compared with the control group) for balance, for physical coordination and for declarative memory retrieval.

Further research, including research using an active control intervention, would be needed to pinpoint the theoretical causes of the improvements obtained. Nonetheless, given the minimal cost and considerable ease of access of the intervention, it provides a promising approach to improving the overall cerebellar-related function, protecting against subsequent balance problems, and may also benefit declarative memory in older adults.

## Author Contributions

Both authors contributed to all aspects of the empirical work, the data analysis and the article writing. ZG had a stronger focus on the empirical work, and RIN had a stronger focus on design and theoretical interpretation.

## Conflict of Interest Statement

Zing Performance Inc. provided free registration on the Zing Programme for the intervention participants and £30 in acknowledgment for those completing the intervention. Neither author received financial support from Zing Performance Ltd.

## Footnotes

^1^www.Zing.com
